# Diversity climate and discrimination at German universities: a cross-sectional study among students in health-related degree programs

**DOI:** 10.1186/s12909-026-09806-3

**Published:** 2026-07-07

**Authors:** Saskia Hanft-Robert, Adekunle Adedeji, Adrian Junger, Marlene Wember, Demet Dingoyan, Ines Heinen, Maja-Lina Böcher, Sidra Khan-Gökkaya, Martin Härter, Franka Metzner-Guczka

**Affiliations:** 1 Department of Medical Psychology, University Medical Center Hamburg Eppendorf, Hamburg, Germany; 2 Division of Health Systems and Public Health, Faculty of Medicine and Health Sciences, Stellenbosch University, Stellenbosch, South Africa; 3 Faculty of Social Work and Childhood Education, Hamburg University of Applied Sciences, Hamburg, Germany; 4 Department of Medical Sociology, University Medical Center Hamburg Eppendorf, Hamburg, Germany; 5 University Medical Center Hamburg-Eppendorf, Hamburg, Germany

**Keywords:** Diversity climate, Discrimination, Student, Health care, Education, University

## Abstract

**Background:**

Diversity is essential in higher education, particularly in health-related programs (e.g., medicine, psychology, and other health professions). Inclusive and non-discriminatory learning environments are crucial for promoting equity and preparing future health care professionals to care for diverse populations. However, empirical evidence on students’ perceptions of diversity in German universities remains limited. This study aimed to 1) assess students’ perceptions of the diversity climate in their health-related degree programs and university contexts and 2) examine differences in psychosocial and academic variables between students with and without experiences of discrimination.

**Methods:**

A national cross-sectional online survey was conducted between January and April 2024 among students in health-related degree programs at German universities. Measures included sociodemographic and educational characteristics, students’ perceptions of diversity climate (across three dimensions: age, ethnicity, gender), experiences of discrimination, diversity- and equity-oriented beliefs, sense of belonging to the university, study engagement, general and academic self-efficacy, and general and study-related stress. Descriptive analyses were conducted as well as independent t-tests to examine differences between groups.

**Results:**

A total of 987 students from 83 universities across all 16 federal states participated. Students’ perceptions of the diversity climate were moderately to highly positive across age (M = 3.91/5, SD = .59), ethnicity (M = 3.63/5, SD = .65), and gender (M = 3.83/5, SD = .59). While the majority reported no personal discrimination at their university, 28% experienced discrimination within the past two years, most commonly due to gender (42%), physical appearance (23%), mental health (20%), or racial reasons (13%). Additionally, 45% had witnessed discrimination. Students with personal experiences of discrimination reported significantly less positive perceptions of the diversity climate, lower academic self-efficacy, study engagement, and sense of belonging at the university, higher stress, and stronger diversity- and equity-oriented beliefs. Across the full sample, 61% indicated that teaching materials do not adequately represent diverse groups, and 50% perceived lecturers as insufficiently responsive to discrimination.

**Conclusion:**

Despite generally positive attitudes toward diversity, the students' high rates of experiences of discrimination and their associations with psychosocial and academic variables highlight the need for systematic efforts to promote inclusive learning environments. Targeted interventions, such as diversity training for staff and students, improved representation in teaching materials and curricula, and stronger institutional policies, are essential to promote equity and well-being in health-related education. By using a nationwide scope, focusing on health-related degree programs, and conducting a multidimensional assessment of students’ perceptions of diversity climate, this study offers important evidence to inform such efforts and addresses a notable gap in the literature.

**Supplementary Information:**

The online version contains supplementary material available at 10.1186/s12909-026-09806-3.

## Introduction

Diversity refers not only to the degree of difference or variety within a group or community but also to the recognition of human heterogeneity as an inherent characteristic of societies and the principle of equality as a fundamental human right [1–4]. How institutions understand and operationalize diversity directly shapes whose interests and needs are represented, acknowledged and addressed. Insufficient consideration of diversity can lead to decisions that disproportionately disadvantage individuals from underrepresented groups, as their needs or interests may not be adequately reflected in existing institutional procedures or workplace norms [5]. Definitions and conceptualizations of diversity vary across contexts, disciplines and fields of research. In the present study, we adopt a perspective commonly used in diversity and higher education research, according to which diversity is assessed by the extent to which programs or institutions eliminate barriers and ensure that the interests and perspectives of diverse groups are meaningfully represented [5]. From this perspective, diversity encompasses not only the presence of differences among individuals and groups but also the extent to which institutions create equitable and inclusive environments.

In healthcare, the ethical obligation to oppose discrimination is explicitly anchored in professional standards. According to the Declaration of Geneva World Medical Association, [6], members of the medical profession promise to oppose discrimination based on age, disease or disability, creed, ethnicity, gender, nationality, political affiliation, race, sexual orientation, or social standing. In Germany, the General Equal Treatment Act (Allgemeines Gleichbehandlungsgesetz, AGG) is intended to provide legal protection against discrimination based on six diversity characteristics: age, gender, ethnicity, disability, sexual orientation, and religion. However, the mere existence of legal, professional, or ethical frameworks does not, in itself, prevent discriminatory practices. In reality, experiences of unequal treatment continue to be reported across healthcare settings [7–9]. In the medical field in particular, discrimination is often embedded in complex interpersonal, institutional, and structural systems that may make identification, reporting, and legal consequences difficult. Moreover, discriminatory practices and exclusionary experiences can also arise from other, legally unprotected characteristics such as socioeconomic status, health status, marital or parental status, or physical appearance [10].

A growing body of international research and first national studies in Germany demonstrate that discrimination, which manifests itself in various forms and is based on a range of often interrelated diversity characteristics, is widespread within healthcare systems [7–9, 11, 12]. Service users as well as healthcare professionals – including nurses, physicians, and administrative, cleaning and security staff – report experiences of discrimination and stigmatization, frequently in intersecting forms [8, 9, 13–17]. For example, Hamed et al. [8] conducted a systematic review of 213 primary studies on how racism is discussed and produced in the process of delivering, accessing and receiving healthcare across various national contexts up to 2020. Mostafa et al. [15] identified 21 primary international studies on healthcare professionals’ experiences of workplace racism published between 2000 and 2024. In a nationally representative survey in France (*N* = 21,761), Rivenbark and Ichou [16] found the highest rates of both reported discrimination within healthcare and foregone care in the past 12 months among women, immigrants from Africa or Overseas France, and Muslims, whereby overall the survey-weighted prevalence of reported discrimination in healthcare settings was 3.9%.

Evidence from Germany mirrors these findings. A representative German survey of 1,007 individuals found that 26.4% of those who had experienced discrimination had done so in the healthcare system in the past 24 months, most commonly related to disability, chronic illness, transgender identity, and weight [18]. In a survey conducted in 272 German hospitals, the most frequently reported reasons for discrimination-related complaints were ethnicity/migration background or racist attribution, body weight, disability, and chronic illness [11].

To counteract such inequalities and appropriately address the diversity of both service users and (healthcare) staff, it is essential to raise awareness among future healthcare professionals and ensure they acquire the necessary competencies during their university education through appropriate teaching formats. Current and future healthcare professionals should be adequately trained and professionalized to meet the needs of diverse service user groups. Higher education institutions, such as universities, play a crucial role in fostering diversity-related competencies by integrating appropriate educational approaches into health-related degree programs (e.g., medicine, psychology, and other health professions). For instance, developing the ability to recognize discriminatory behaviors and structural barriers often requires a shift in perspective, which can be facilitated through diversity-sensitive teaching approaches. Such approaches have been shown not only to improve students’ confidence in working with diverse service user groups but also to strengthen their sense of belonging and institutional inclusion [19]. Yet, diversity and diversity-sensitive care often remain insufficiently embedded in healthcare curricula. Medical students frequently report that their training does not adequately prepare them to care for diverse service user populations [20] or describe a derogatory discourse about humanities and social science disciplines within their medical curriculum, as well as the limited level of diversity competence among teachers in their medical education curriculum [21]. In contrast, international organizations such as the Lancet Commission on Culture and Health or the International Association for Medical Education (AMEE) call for the inclusion of diversity characteristics, such as ethnicity [22], in medical education. Dogra, Reitmanova & Carter-Pokras [23] published 12 tips for teaching diversity and embedding it in the medical curriculum, addressing institutional policies; the development of relevant content, outcomes, and appropriate delivery methods; faculty development; and the assessment of policies, delivery, and learned outcomes.

Beyond curricular content, diversity must also be addressed within university structures and institutional cultures, including student and faculty composition, admissions processes, accessibility, and teaching materials [24]. For instance, the diversity of (future) healthcare professionals itself has been identified as a key factor in reducing discrimination within healthcare systems [25]. While universities cannot be entirely free of discrimination [26], their commitment to promoting diversity significantly shapes students’ experiences. Beyond formal policies and structures, universities communicate social norms through diversity statements, institutional priorities, teaching practices, and the behavior of faculty and staff, shaping how diversity, inclusion, and discrimination are perceived and addressed within the academic environment.

Students’ experiences of discrimination and their perceptions of institutional diversity climate are closely associated with stress, well-being, and a sense of belonging [27], which, in turn, can predict academic performance and dropout intentions [28]. Diversity climate is understood as a relatively stable group-level construct that refers to how diversity is perceived at the interpersonal level, in the interactions of diverse members of the institution, and on the organizational level, including the values and norms communicated and perceived within an institution regarding diversity [3, 4, 29].

Although several countries have long implemented comprehensive strategies to promote diversity within higher education and (postgraduate) medical training (e.g., [30–32]), Germany remains at an early stage in this development [24, 33]. Nonetheless, institutions that train healthcare professionals in Germany are increasingly being held accountable. In 2018, the German Association for Medical Education (GMA) published a position paper on cultural competencies and global health in medical education, calling not only for the provision of specific courses on cultural competence and global health but also for integrating these topics into the standard curricula of health-related degree programs [34]. More recently, Gerhards et al. [33] called on medical education institutions to actively combat racism at all levels of training and practice. These efforts include establishing guidelines, target agreements, and initiatives to strengthen diversity competence among university members [35, 36].

Despite these developments, empirical evidence from Germany remains limited on students’ experiences of diversity in higher education; their personal experiences and observations of discrimination; the associations between discrimination and academic and psychosocial outcomes; and their perceptions of diversity-related initiatives, particularly within health-related degree programs [3, 37–40]. At the same time, diversity-sensitive training is particularly important given the diversity of the service users who will be treated by the future healthcare professionals. Compared with students in higher education more broadly, students in health-related degree programs are preparing for professions that are often emotionally demanding. In addition to dealing with physical and psychological suffering, pain, and existential questions of life and death, they must navigate complex interactions with service users, colleagues, and supervisors. These interactions are often further complicated by power imbalances, dependencies, and structural barriers to accessing healthcare. Therefore, topics such as self-care, empowerment, and reflection on one’s own actions should be systematically incorporated into the training from the very beginning. To date, however, little is known about the extent to which this has already been implemented in university curricula and structures in Germany.

Thus, the present nationwide study aims to assess the diversity climate at higher education institutions, such as universities, from the perspective of students enrolled in health-related degree programs using a multidimensional approach. In addition, it investigates students’ personal experiences and observations of discrimination and explores differences in academic and psychosocial outcomes between students with and without personal experiences of discrimination. The following key research questions should be addressed:

- RQ1. How do students enrolled in health-related degree programs in Germany perceive the diversity climate at their higher education institutions across different dimensions of diversity (i.e., age, gender, ethnicity)?
- RQ2. To what extent do students report personal experiences of discrimination and observations of discrimination within their higher education institutions?
- RQ3. Are there differences in academic outcomes (i.e., academic self-efficacy, stress due to studies, sense of belonging, study engagement) between students who have personally experienced discrimination and those who have not?
- RQ4. Are there differences in psychosocial outcomes (i.e., general self-efficacy and general stress) between students who have personally experienced discrimination and those who have not?

## Methods

The present study was conducted between January and April 2024 at the Department of Medical Psychology at the University Medical Center Hamburg-Eppendorf and employed a national cross-sectional online survey design. Ethical approval was obtained from the Ethics Committee of the University Medical Center Hamburg-Eppendorf on 19 September 2023 (LPEK-0671). Prior to participation, respondents received standardized information outlining the study’s objectives, procedures, and voluntary nature, as well as the anonymous handling of data. An incentive in the form of a voucher lottery was offered upon completion of the questionnaire. Informed consent was obtained electronically from all participants at the beginning of the survey.

### Development of the questionnaire

We developed the online questionnaire in a multistage process. Based on the literature review, relevant themes were selected and discussed within our interdisciplinary research group, which included experts from medical education, public health, migration studies, medical sociology, psychology, psychotherapy, and educational science. Guided by these themes, we used items from previous studies and existing validated scales, where available, and developed study-specific items when suitable measures were not available.

A first version of the questionnaire underwent three stages of pretesting. In the first stage, we conducted cognitive interviews with three master’s psychology students from three different universities. Inquiring and paraphrasing techniques were used to assess the clarity and comprehensibility of the questions and response scales [41]. In the second stage, we sought feedback from five experts in diversity research on the questionnaire items and overall structure. Based on their input, we revised the questionnaire and conducted a pretest with eight students from various health-related degree programs (e.g., medicine, psychology) during the final stage of survey development. We checked the technical functionality, the filter question flow, and the formatting and corrected any errors [41]. Additionally, we optimized the questionnaire display for mobile devices. The questionnaire was available in German and English. Translations into German or English were done using the Translation, Review, Adjudication, Pretest, and Documentation (TRAPD) approach of questionnaire translation [42, 43].

The final survey included four main sections: collecting sociodemographic data; examining discrimination (pesonally experienced and observed); measuring students’ perceptions of the diversity climate; assessing psychosocial and academic aspects; and evaluating diversity initiatives at participants' universities. These sections align with Hagelskamp’s [3] conceptual model of diversity climate. After consenting to participate, respondents were presented with a brief definition of diversity and then asked to share their views on the topic. The sociodemographic section collected information on age, gender, ethnicity, academic level, and other variables relevant to the intersectionality of diversity and discrimination. Next, participants were asked about their personal experiences of discrimination and the instances they observed. Participants also evaluated their perceptions of campus diversity. Subsequently, they were asked about their well-being and academic aspects, including study engagement, general and study-related stress, sense of belonging to the university, and general and academic self-efficacy. The final section focused on evaluating diversity measures at their institutions, including the availability of support services (e.g., counseling and anti-racism resources) and the perceived sensitivity of faculty, staff, and teaching to issues of diversity and discrimination. At the end of the survey, participants were invited to provide open-ended feedback, suggest diversity-related improvements at their university, and share any additional comments. Participants could skip any questions, including those related to sensitive topics such as sociodemographic data or experiences of discrimination.

### Measurements

#### Sociodemographic variables

The survey included single- or multiple-choice questions on age, gender identity, sexual orientation, highest professional qualification, parental higher-education degree, social class, participants’ and parents’ countries of birth, ethnicity, belonging to an ethnic minority, and religion. Most items were adapted from the Diversity Minimal Item Set (DiMIS) by Stadler and colleagues [44]. For all questions, participants could provide their own self-description in addition to predefined single- or multiple-choice answers. Additionally, participants were asked about any care responsibilities as well as the number and types of physical and mental health impairments they experience.

#### Current education

Regarding participants’ current education, we asked single- or multiple-choice questions about their field of study, current semester, academic performance, and university affiliation. We also assessed their intentions to change or end their studies, or to change universities, on a five-point Likert scale ranging from *“1* = *I have never thought about it”* to *“5* = *I often think about it”* [45].

#### Discrimination

We developed two items to assess personal experiences of discrimination and observed instances of discrimination based on the DiMIS [44]. We asked, *“In the last 24 months, have you personally experienced discrimination for the following reasons at your university?”* and “*In the last 24 months, have you observed any situations at your university in which one or more people were discriminated against for the following reasons?”* As answer options, we provided 15 diversity characteristics, including the six dimensions covered by the AGG (i.e., age, ethnicity, disability, gender, religion, and sexual orientation), based on the Hamburg Discrimination Questionnaire (Hamburger Diskriminierungsfragebogen; HDF) by Dingoyan et al. [46]. Participants could also add other reasons for discrimination in an open-response field.

#### Diversity climate

We assessed participants’ perceived diversity climate using an adapted and extended version (we deleted 5 items and added 1) of the intergroup interactions domain of the School Climate for Diversity – College Scale (SCD-C) by Byrd [47, 48]. For economic reasons and the survey's length, we focused only on the intergroup interactions domain rather than using the entire SCD-C scale. The intergroup interactions domain aligns with the conceptual framework of this study, in which diversity climate encompasses both interpersonal interactions among diverse institutional members and organizational-level norms and values [3]. The campus racial socialization domain [48] was excluded due to survey length constraints and its specific focus on the U.S. higher education context.

The original SCD-C scale focuses only on ethnicity. In the present study, we expanded the scale and included age and gender. Based on feedback from experts, cognitive interviews with students, and pre-tests, we decided to include these two characteristics because they were considered easier to perceive or attribute in everyday social interactions than some other diversity characteristics, such as socioeconomic background. Importantly, we do not assume that age, gender, or ethnicity can always be accurately identified from appearance alone, and that this assumption, for instance, may lead to misgendering persons. Characteristics such as disability, religion, sexual orientation, or socioeconomic background may likewise be perceived, disclosed, or identified in certain contexts. However, we had to limit the number of diversity characteristics, as including more would have substantially increased survey length and participant burden, as each dimension required separate assessment. Consequently, the present study focused on age, ethnicity, and gender as dimensions of diversity. Each characteristic was assessed with 15 items, resulting in a 45-item diversity climate scale.

The psychometric properties of the adapted scale were examined in a validation study prior to the present study, using the same dataset. The results will be published elsewhere. Confirmatory factor analyses confirmed the five-factor structure of the Intergroup Interactions domain separately for ethnicity-, age-, and gender-based versions (ethnicity: CFI = 0.96, TLI = 0.95, RMSEA = 0.055, SRMR = 0.041; age: CFI = 0.96, TLI = 0.95, RMSEA = 0.049, SRMR = 0.043; gender: CFI = 0.97, TLI = 0.96, RMSEA = 0.047, SRMR = 0.035), with composite reliability values ranging from 0.60 to 0.88. However, convergent validity was not fully established across all subscales and versions.

The intergroup interactions domain was measured by five dimensions: 1. quality of interaction, 2. frequency of interaction, and 3. stereotyping, which operationalize the interpersonal level, while 4. equal status and 5. support for positive interaction represent the organizational level of diversity. In our study, the five dimensions were measured with three items each. For each item, we presented the item stem (e.g., *"Students … trust each other."*) and displayed the three diversity characteristics below the stem: *“…of different ages…”, “…of different ethnic backgrounds…”*, and *“…of different genders”*. Participants rated each characteristic on a 5-point Likert scale, ranging from *“1* = *not at all true” to “5* = *completely true”*, with higher sum scores indicating more positive views of the different dimensions.

- 1. *Quality of Interaction:* This dimension assesses the quality of interpersonal interactions among diverse groups, focusing on how well these groups understand each other, trust one another, and get along (e.g., *“People of different [ages] [ethnic backgrounds] [genders] have trouble getting along with each other.”*). The Cronbach’s alpha reliability scores, which measure the internal consistency of a test or scale [49], were 0.72 for age, 0.78 for ethnicity, and 0.72 for gender in the present sample.
- 2. *Frequency of Interaction:* This dimension assesses how often students from diverse backgrounds engage with each other outside of classes, collaborate in academic settings, and study together (e.g., *“Students of different [ages] [ethnic backgrounds] [genders] hang out together.”*). The Cronbach’s alpha reliability scores were 0.69 for the age subscale, 0.80 for ethnicity, and 0.79 for gender in the present sample.
- 3. *Equal Status:* This dimension assesses whether students perceive themselves as being treated fairly and equally by university staff and faculty members (e.g., *“At my university, faculty are fair to students of all [ages] [ethnic backgrounds] [genders]”*). The Cronbach’s alpha reliability scores were 0.83 for the age subscale, 0.88 for ethnicity, and 0.85 for gender in the present sample.
- 4. *Support for Positive Interaction:* This dimension examines how effectively faculty members foster positive interactions among students from diverse backgrounds and whether university policies and teaching practices effectively convey the importance of diversity (e.g., *“Faculty encourage contact between students of all [ages] [ethnic backgrounds] [genders]”*). The Cronbach’s alpha reliability scores were 0.80 for the age subscale, 0.78 for ethnicity, and 0.75 for gender in the present sample.
- 5. *Stereotyping:* This dimension examines whether students and faculty hold stereotypical views or biases about specific groups and to what extent these groups are stereotypically represented (e.g., *"People of different [ages] [ethnic backgrounds] [genders] are represented in stereotypical ways in textbooks and lectures."*). The Cronbach’s alpha reliability scores were 0.60 for the age subscales, 0.70 for ethnicity, and 0.66 for gender in the present sample.

#### Diversity at the institution

To assess how diversity is integrated into the educational context, several items were self-developed or adapted from previous studies. We asked about the availability and utilization of support services (e.g., for gender, age, care work, ethnicity) [50]. Further items asked about the extent to which different diversity characteristics are integrated into the curriculum (ranging from *“1* = *not adequately covered”* to *“4* = *too comprehensively covered”*) [51], and about the perceived diversity among students (ranging from *“1* = *not diverse”* to *“4* = *very diverse”)* [52]. Moreover, two items regarding the lecturers (i.e., *“Teachers are sensitive to discrimination and work to counteract it”,* 5-point Likert Scale ranging from *“1* = *far too little”* to *“5* = *far too much”)* and teaching materials (i.e., *“The teaching materials and examples depict people with diverse characteristics and do not portray them in a stereotypical manner”,* 5-point Likert Scale ranging from *“1* = *far too little”* to *“5* = *far too much”)* were included [50]. Four items asked about students’ diversity competencies gained through their study programs (e.g., *“My program helped me become aware of the consequences of my own prejudices.”*), which could be rated on a 5-point Likert Scale ranging from *“1* = *fully disagree”* to *“5* = *fully agree”)* [53].

#### Diversity and equity-oriented beliefs

To assess participants' perceptions of diversity and equity-oriented beliefs and their associated benefits in social and academic contexts, an adapted version of the Pro-Diversity Beliefs Scale [54] by Hagelskamp et al. [4] was employed, comprising five items that can be rated on a 5-point Likert Scale ranging from *“1* = *does not apply at all” to “5* = *applies completely”*. The original items were slightly adapted for the study context (e.g., *“When student groups are highly diverse, they learn to better cope with future societal challenges.”).* Higher sum scores on this scale reflect a robust belief in the positive impacts of diversity, whereas lower scores indicate a more negative perception of it. The Cronbach’s alpha reliability score for the scale was 0.87 in the present sample.

#### Study engagement

The Ultra-Short Measure for Work Engagement (UWES-3; [55, 56]) was used to assess students' engagement in their studies. The scale consists of three items, each measuring one particular aspect of work engagement: vigor, dedication, and absorption (e.g., *“While I work for my studies, I feel bursting with energy”*). Items could be answered on a 7-point Likert Scale ranging from *“1* = *never”* to *“7* = *always”.* Higher scores on this scale indicate greater work engagement. The Cronbach’s alpha reliability score for the scale was 0.84 in the present sample.

#### Belonging to the university

Belonging to the university was measured using an adaptation of the School Belonging Scale (SBS; [57]. The item stem clarified the reference to the university context (i.e., *“Thinking about your university, how much do you agree with the following statements?”*). The scale comprised one positively worded item (i.e., *“I feel that I belong at my university”*) and three negatively worded items (e.g., *“I feel like an outsider at my university”*). All items were rated on a 7-point Likert scale ranging from *“1* = *strongly disagree” to “7* = *strongly agree”.* Lower scores on this scale indicate experiences of exclusion, whereas higher scores reflect a strong sense of belonging. The Cronbach’s alpha reliability score for the scale was 0.87 in the present sample.

#### General self-efficacy

The General Self-Efficacy Short Scale-3 (GSE-3; [58, 59]) was administered to assess participants' global confidence in their ability to navigate life’s challenges with three items (e.g., *“I can rely on my own abilities in difficult situations”),* which can be answered on a 5-point Likert Scale ranging from “*1 =* *does not apply at all”* to “5 = *applies completely*.” Higher values on this scale demonstrate a strong belief in one’s ability to overcome obstacles and effectively solve problems, while lower scores indicate a lack of faith in personal problem-solving abilities. The Cronbach’s alpha reliability score was 0.82 in the present sample.

#### Academic self-efficacy

The General Academic Self-Efficacy Scale (GASE; [60]) assessed participants' self-confidence in performing academic tasks and their belief in their ability to achieve study-related goals (e.g., *“Generally, I can solve difficult academic problems if I try hard enough”*), that can be answered on a 5-point Likert Scale ranging from *“1* = *strongly disagree at all”* to *“5* = *strongly agree”*, was employed. Higher scores suggest a strong sense of capability regarding academic skills, while lower scores indicate uncertainty about one’s ability to meet academic goals. The Cronbach’s alpha reliability score was 0.75 in the present sample.

#### General stress

General perceived stress was assessed with two items based on a study conducted by Schmidt et al. [61], *“During the last 4 weeks, how often did you feel stressed and tense?”* which could be answered on a 5-point Likert scale ranging from *“1* = *never”* to *“5* = *daily”.* The second item, *“How would you describe your life at the moment? As…”* could be rated on a 5-point Likert scale ranging from *“1* = *not at all stressful”* to *“5* = *highly stressful”.* Higher sum scores indicate higher levels of general stress. The Cronbach’s alpha reliability score was 0.84 in the present sample.

#### Academic stress

Academic stress related to participants’ studies was measured using the item, “*During the last 4 Weeks, how stressed did you feel because of your studies?”*, which could be answered on a scale from “*0 = not at all stressed”* to *“100 = completely stressed”*, with higher scores indicating greater academic stress [61].

### Participants’ recruitment and data collection

Participation was open to all students aged 18 or older who were enrolled at a higher education institution in Germany at the time of the survey and were studying a health-related degree program as their primary, secondary, or supplementary field of study. Health-related degree programs were defined as those that focus on the biological, social, physical, and/or psychological aspects of health and illness and whose graduates are qualified for professional roles in the healthcare sector. Examples of such programs included medicine, psychology, social work, and public health. No additional inclusion or exclusion criteria were applied.

Convenience and snowball sampling approaches were used for recruitment. Nine federal student councils were contacted, and the survey was distributed through 14 Facebook groups and personal networks. Additionally, 94 Universities of Applied Sciences and approximately 400 student councils from around 70 universities were contacted. Five weeks after the survey launched, follow-up emails were sent. Incentives, including vouchers worth €15, €50, and €100, were raffled among the first 500 participants who provided their student email address at the end of the survey. The costs for the vouchers were fully covered by the Department of Medical Psychology at the University Medical Center Hamburg-Eppendorf. Following the snowball sampling approach, participants were asked to share the study link with other students at the end of the survey. Data were collected between January and April 2024 using the online survey tool SoSci Survey (https://www.soscisurvey.de/).

### Data analysis

Before the data analysis, a comprehensive series of preparatory steps was undertaken to ensure data cleanliness and integrity. The initial sample comprised *N* = 1,237 participants. However, *n* = 7 individuals were excluded because they opted out of the data privacy policy. Additionally, *n* = 11 participants were removed from the dataset because they reported studying non-health-related subjects. Lastly, cases with 60% or more missing data (in all numeric variables of the original dataset) were excluded from the analyses (*n* = 232), including participants who did not pass an attention check in the questionnaire. Consequently, the final sample consisted of *N* = 987 participants. Reverse-coded items from the School Belonging Scale (SBS; [57]) and the subscales Quality of Interaction and Stereotyping of the Diversity Climate Scale [47, 48] were appropriately recoded, and grouping variables were computed (at least one discrimination experience vs. no discrimination experience). Following these data preparation steps, descriptive analyses were conducted as well as independent *t*-tests to examine differences between participants who have experienced discrimination and those who have not. Effect sizes for group differences were estimated using Hedges’ *g* with 95% confidence intervals due to unequal group sizes. All analyses were performed using IBM SPSS Statistics Version 28.0.1.1.

## Results

### Sociodemographic characteristics

On average, the participants were 25.45 years old (SD = 5.67, range = 18–60 years) (see Table 1). The majority of them identified as female (77.5%), which corresponds to the proportion of female students in fields such as human medicine (64.6%), psychology (76.5%), and health science in general (76.8%) across Germany [62]. About 70.6% considered themselves part of the (upper-)middle class. Most participants identified as heterosexual (66.6%), were born in Germany (81.2%), did not consider themselves part of an ethnic minority or racialized group (85.7%), and identified as *White* (68.2%). Most were Christians (37.1%) or atheists (24.5%). In total, 49.6% indicated at least one health impairment, with mental health conditions (29.6%), chronic physical health issues (10.3%), and visual impairment (9.5%) being the most frequent ones (see Table A in the appendix).

**Table 1:** Sociodemographic data of the sample of students in health-related degree programs in Germany (*N* = 987)

	*n* (%)/range	*M (SD)*
Age (in years)	18–60	25.45 (5.67)
Gender Identity^a^		
Female	765 (77.5)	
Cis	113 (14.8)	
Trans	3 (0.4)	
Male	180 (18.2)	
Cis	38 (21.1)	
Trans	3 (0.4)	
Non-binary	37 (3.7)	
Trans	23 (2.3)	
Endo	12(1.2)	
Questioning	11 (1.1)	
Prefer to self-identify^b^	2 (0.2)	
Sexual Orientation^a^		
Heterosexual	657 (66.6)	
Bisexual	195 (19.8)	
Homosexual	65 (6.6)	
Asexual	38 (3.9)	
Pansexual or Omnisexual	70 (7.1)	
Prefer to self-identify^b^	35 (3.5)	
Parental Higher Education Degree		
Yes (at least one parent)	608 (61.6)	
No	376 (38.1)	
Social class		
Lower class	218 (22.1)	
Middle class	397 (40.2)	
Upper-middle class	300 (30.4)	
Upper class	53 (5.4)	
None of the above	2 (0.2)	
Participants' Place of Birth		
Germany	801 (81.2)	
Abroad	90 (9.1)	
Parents' Place of Birth		
Both parents were born in Germany	687 (69.6)	
At least one parent was born outside Germany	201 (20.4)	
Belonging to an ethnic minority		
Yes	121 (12.3)	
No	846 (85.7)	
Ethnicity^a^		
White	673 (68.2)	
Asian-German	33 (3.3)	
BIPoC (Black, Indigenous, and People of Color)	23 (2.3)	
Russia-German	20 (2.0)	
Eastern European-German	20 (2.0)	
Arabic-German	19 (1.9)	
Turkish-German	16 (1.6)	
Black	15 (1.5)	
Polish-German	13 (1.3)	
Afro-German	13 (1.3)	
Formerly Yugoslavian-German	11 (1.1)	
Latin-American-German	7 (0.7)	
Sinti and Roma	1 (0.1)	
Prefer to self-identify^b^	88 (8.9)	
Religion and world beliefs^a^		
Christianity	359 (37.1)	
Islam	27 (2.8)	
Buddhism	9 (0.9)	
Hinduism	7 (0.7)	
Judaism	1 (0.1)	
Atheistic	237 (24.5)	
Agnostic	115 (11.9)	
No specific religion	199 (20.6)	
Prefer to self-identify^b^	13 (1.3)	
Care responsibilities^a^		
For an adult	52 (5.3)	
For a child (< 18 years)	48 (4.9)	

^a^multiple answers possible

^b^open answer

We reached students from different disciplines, with medical professions (26.5%), health and rehabilitation science (18.5%), and psychology/psychotherapy (15.5%) being the largest groups (see Table B in the appendix). On average, students were in their 5th semester (M = 4.95, SD = 3.44), ranging from the 1st to the 21st semester. Students from across the country participated in our study, with most based in North Rhine-Westphalia (13.4%), Baden-Württemberg (11.8%), and Bavaria (10%), which were also the three federal states in Germany with the highest overall number of students (25.7%, 12.2%, and 13.8%) in the winter semester 2023/2024 [63]. Most students have never or rarely considered changing their study program (85.3%), ending their studies (87.9%), or transferring to another university (85.3%).

### Diversity climate

Across all five subscales, mean scores generally indicated a moderately to highly positive perceived diversity climate across the three selected diversity characteristics, age (M = 3.91, SD = 0.59), ethnicity (M = 3.63, SD = 0.65), and gender (M = 3.83, SD = 0.59; see Table 2). Regarding the sub-scales (see Table C in the appendix), quality of interaction was rated relatively positively, with the highest mean score for gender (M = 4.05, SD = 0.68), similarly for frequency of interaction with a mean score for gender of M = 4.07 (SD = 0.75). Perceptions of equal status were consistently high across all diversity characteristics (mean scores between 4.01 and 4.17). In contrast, support for positive interaction received the lowest ratings overall (mean scores ranging from 2.80 to 3.08). Stereotyping scores were moderate and similar across age, ethnicity, and gender (mean scores ranging from 3.81 to 3.88). Overall, gender-related perceptions were rated more positively than those related to age or ethnicity. While most diversity climate subscales demonstrated acceptable to good internal consistency, the stereotyping subscale showed comparatively lower reliability coefficients, especially for age (α = 0.60), suggesting that results for this dimension should be interpreted with caution.

**Table 2:** Group differences between students with and without personal discrimination experience in health-related degree programs in Germany (N = 987)

Variables	Total			None personal discrimination experience (*n* = 687)		At least one personal discrimination experience (*n* = 280)		t(df)	p	Hedges’ g [95% CI]
		M	SD	M	SD	M	SD			
Diversity climate – age^1a^		3.91	.59	3.72	.54	3.34	.60	9.60 (965)	<.001	.68 [.54,.82]
Diversity climate – ethnicity^1a^		3.63	.65	3.77	.59	3.31	.66	10.61 (965)	<.001	.75 [.61,.89]
Diversity climate – gender^1a^		3.83	.59	3.97	.53	3.52	.60	10.73 (965)	<.001	.80 [.66,.94]
Diversity and equity-oriented beliefs^1b^		4.16	.78	4.13	.76	4.25	.82	–2.12 (964)	.034	–.15 [–.29, –.01]
Academic self-efficacy^1c^		3.96	.63	4.00	.62	3.87	.65	2.70 (900)	.007	.20 [.05,.34]
General self-efficacy^1d^		3.97	.63	3.99	.62	3.95	.66	.87 (901)	.382	.07 [–.08,.21]
Study engagement^2e^		4.54	1.14	4.63	1.12	4.32	1.18	3.74 (907)	<.001	–.15 [–.29, –.01]
Belonging to the university^2f^		5.2	1.47	5.46	1.34	4.56	1.59	8.71 (907)	<.001	.64 [.49,.79]
Academic stress^3g^		64.27	23.78	63.40	23.96	66.55	23.58	−1.79 (904)	.074	-.13 [-.28,.01]
General stress^1h^		3.6	.88	3.52	.88	3.79	.85	–4.09 (908)	<.001	–.30 [–.45, –.16]

^1^The scale ranges from 1 to 5

^2^the scale ranges from 1 to 7

^3^the scale ranges from 0 to 100

Higher values indicating ^a^more positive view on the diversity climate dimensions (age, ethnicity, gender), ^b^more robust belief in the positive impacts of diversity, ^c^stronger sense of capability regarding academic skills, ^d^stronger belief in personal problem-solving abilities, ^e^greater work engagement, ^f^stronger sense of belonging, ^g^greater academic stress, ^h^higher levels of general stress

### Discrimination experience and observation

**Fig. 1:**
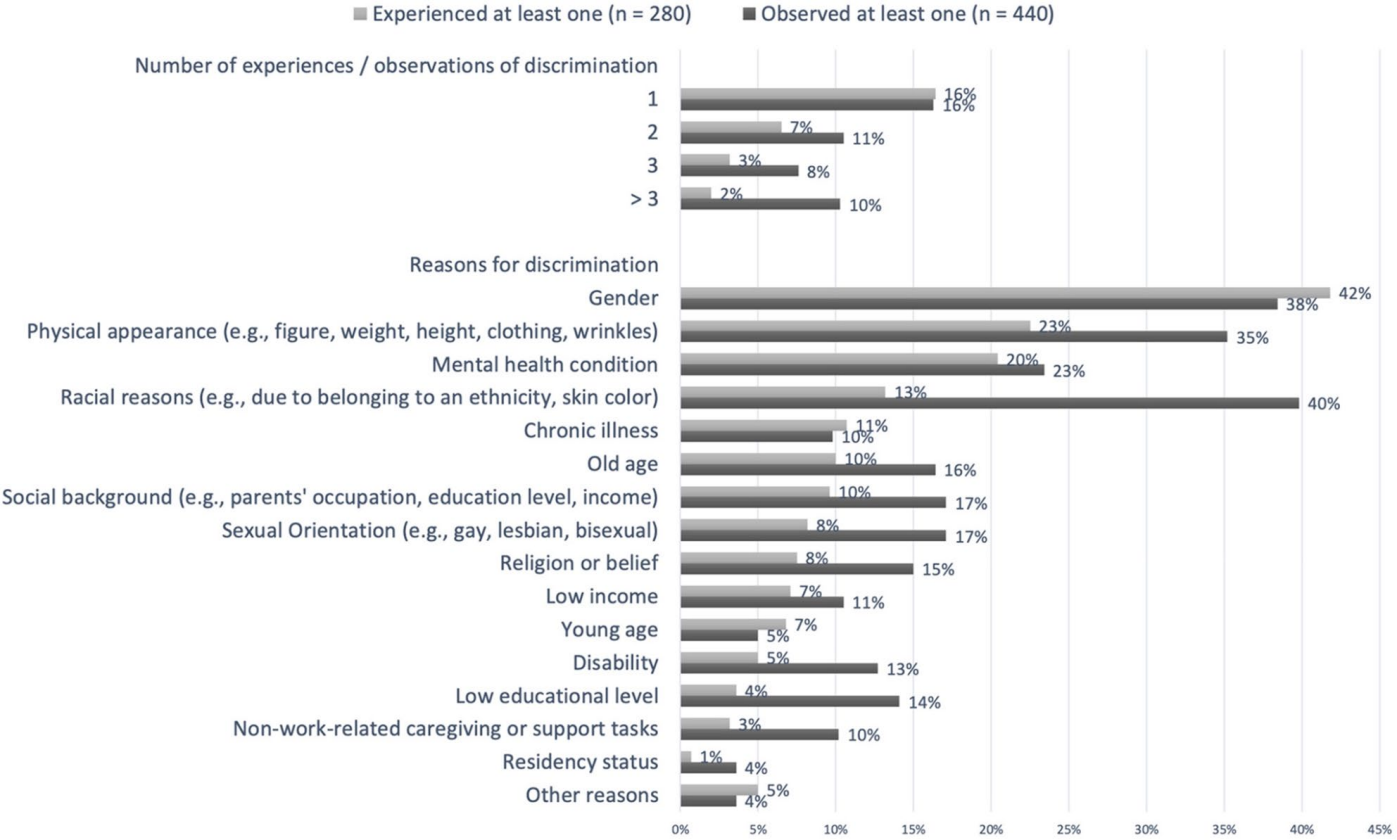
Number of and reasons for experience and observation of discrimination given by students in health-related degree programs in Germany (*N* = 987) Note. For ease of presentation, percentages are reported without decimal places and were rounded to the nearest whole number

Almost one-third of the sample (28.4%) had personally experienced discrimination, most commonly due to gender (42%), physical appearance (e.g., figure, weight, height; 23%), mental health issues (20%), or racial reasons (13%). In total, 45% had observed discrimination within their university context, with racial reasons (40%), gender (38%), physical appearance (35%), and mental health condition (23%) being the most frequently mentioned reasons (see Fig. 1).

### Group differences between individuals with and without personal discrimination experience

#### Perceived diversity climate (age, ethnicity, gender)

To explore differences between individuals who have personally experienced discrimination (28.4%) and those who have not (69.6%), we conducted independent *t*-tests. In this sample, we found significant group differences in perceived diversity climate across all three dimensions (see Table 2). Individuals with at least one personal experience of discrimination reported significantly less positive perceptions of the diversity climate with regard to age (*t*(965) = 9.60, *p* < 0.001), ethnicity (*t*(965) = 10.61, *p* < 0.001), and gender (*t*(965) = 10.73, *p* < 0.001), compared to individuals without discrimination experiences. Effect sizes ranged between medium for age (*g* = 0.68, 95% CI [0.54, 0.82]) and large for ethnicity and gender (*g* = 0.75, 95% CI [0.61, 0.89]; *g* = 0.80, 95% CI [0.66, 0.94]).

#### Diversity and equity-oriented beliefs

Participants who personally experienced discrimination expressed stronger diversity and equity-oriented beliefs than those who did not (*t*(964) = − 2.12, *p* = 0.034). The effect size was small with *g* = –0.15, 95% CI [–0.29, –0.01].

#### Study related variables

Students who personally experienced discrimination reported significantly lower academic self-efficacy (*t*(900) = 2.70, *p* = 0.007), lower study engagement (*t*(907) = 3.74, *p* < 0.001), and lower belonging to the university (*t*(907) = 8.71, *p* < 0.001) compared to students without personal discrimination experience. Regarding study-related stress over the past 4 weeks, students with at least one experience of personal discrimination reported slightly higher stress levels. However, the results were not statistically significant (*t*(904) = − 1.79, *p* = 0.074). Effect sizes ranged between small for academic self-efficacy and study-engagement (*g* = 0.20, 95% CI [0.05, 0.34]; *g* = –0.15, 95% CI [–0.29, –0.01]) and medium for school belonging (*g* = 0.64, 95% CI [0.49, 0.79]).

#### Psychosocial variables

Students who reported at least one experience of personal discrimination reported significantly higher levels of perceived general stress (*t*(908) = − 4.09, *p* < 0.001). Effect size was small (*g* = − 0.30, 95% CI [–0.45, –0.16]). No significant group differences emerged for general self-efficacy (*t*(901) = 0.87, *p* = 0.382); however, self-efficacy was slightly lower among participants with discriminatory experiences (M = 3.95 vs. 3.99).

### Diversity at the university

#### Support services and infrastructure

**Fig. 2:**
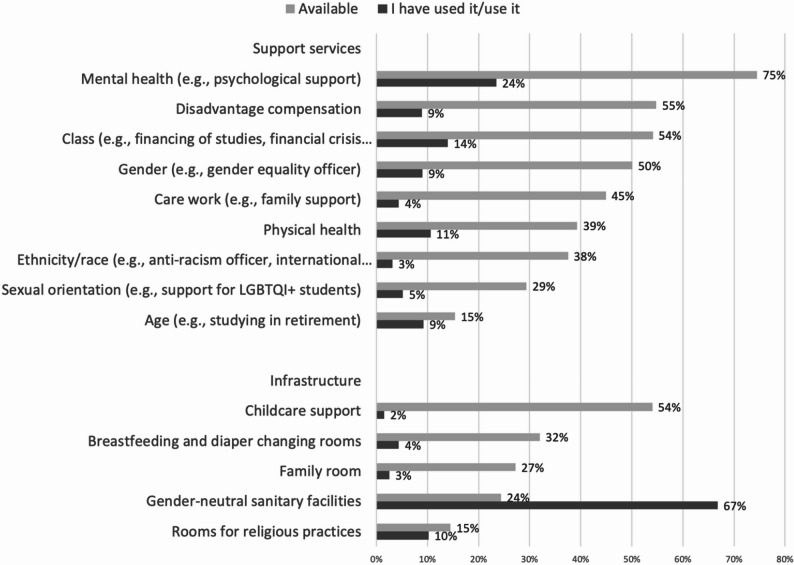
Available and used diversity support services and infrastructure at German universities (*N* = 987) Note. For ease of presentation, percentages are reported without decimal places and were rounded to the nearest whole number

Looking at institutional support services, such as gender equality officers, family support, and psychological counseling, results indicate that these services are rarely used even when available (see Fig. 2). Regarding the universities’ physical infrastructure, 22.6% of respondents reported that campus buildings are not accessible for all individuals, 41.1% indicated that they are only partly accessible, and 36.2% considered them fully accessible.

#### Diversity among students

**Fig. 3:**
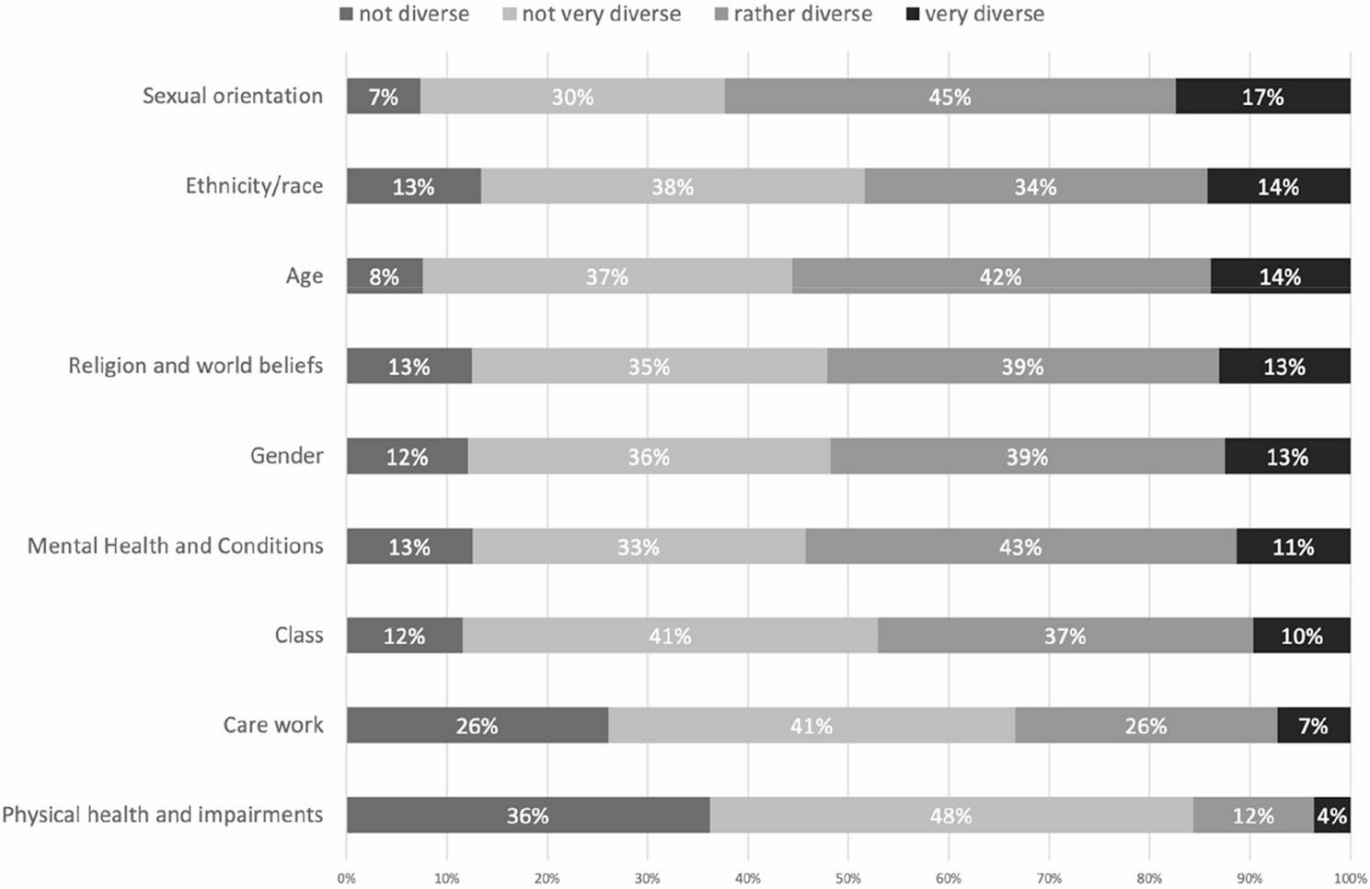
Perceived diversity of students in health-related degree programs in Germany (*N* = 987) Note. For ease of presentation, percentages are reported without decimal places and were rounded to the nearest whole number

Regarding perceptions of student diversity, participants generally viewed the student population as not very or rather diverse across multiple characteristics (M = 2.45, SD = 0.55). Perceived diversity across specific dimensions, i.e., age, gender, ethnicity/race, physical health, mental health, religion, sexual orientation, caregiving responsibilities, and social class, is presented in Fig. 3. Moreover, 22.6% of respondents disagreed that the university publicly presents a realistic portrayal of the diversity of its student population, for instance, in brochures or on its websites. An additional 30.7% partially agreed, while 46.7% agreed that these public representations are realistic.

#### Diversity among lecturers, learning materials and curricula

**Fig. 4:**
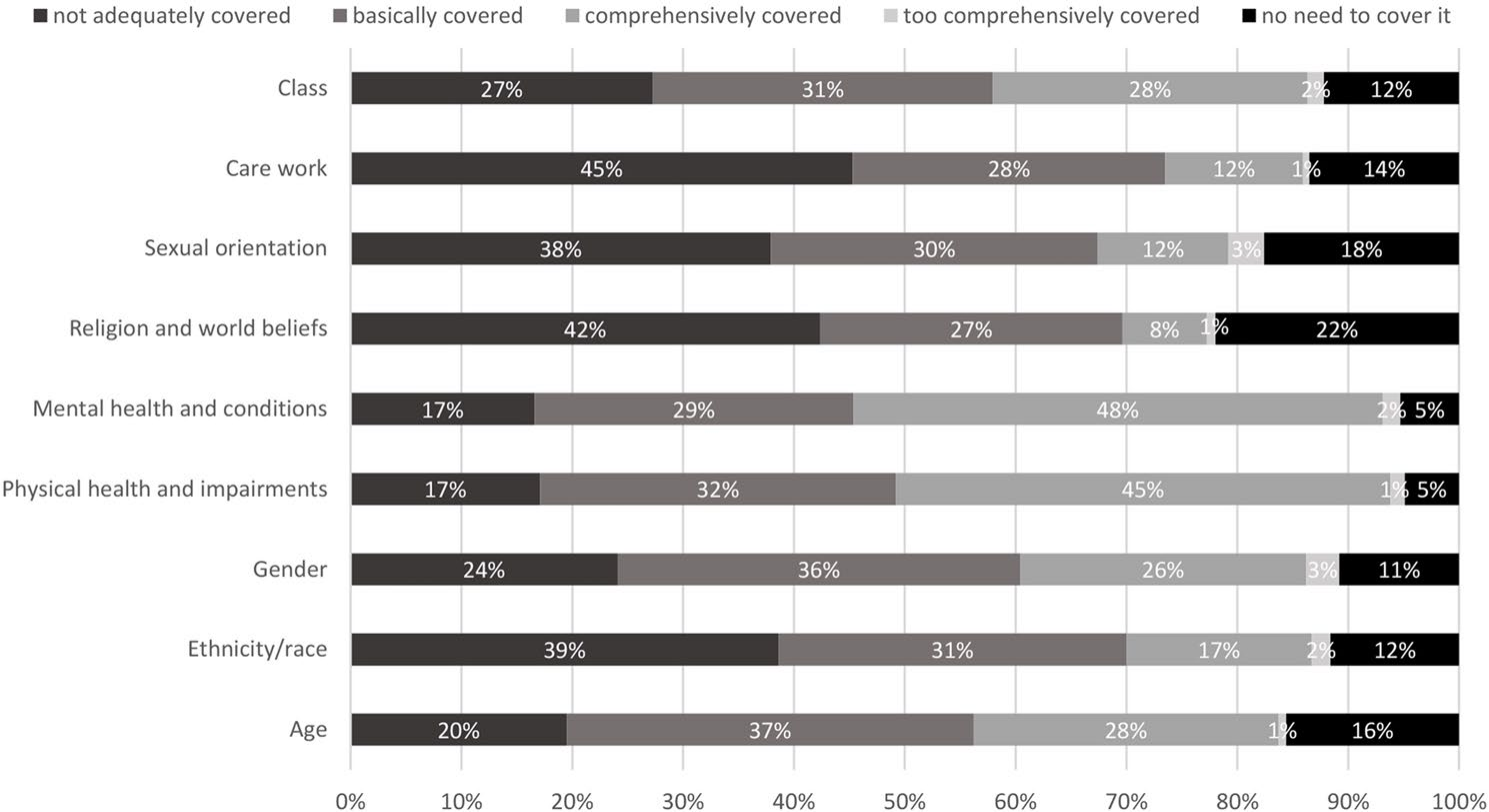
Integration of diversity characteristics within the curricula of health-related degree programs in Germany (*N* = 987) Note. For ease of presentation, percentages are reported without decimal places and were rounded to the nearest whole number

Students were also asked about their perceptions of lecturers and their teaching practices. Nearly half of the respondents (49.9%) indicated that their lecturers are neither sufficiently sensitive to nor actively counter discrimination (M = 2.43, SD = 0.80). Furthermore, 60.5% reported that teaching materials do not adequately represent individuals with diverse characteristics (M = 2.30, SD = 0.78). A detailed overview of how well various diversity characteristics are addressed within participants’ curricula is provided in Fig. 4.

#### Diversity competence

Regarding diversity competencies gained through their study programs, participants on average partially agreed that their studies had encouraged greater respect toward different groups of people (M = 3.39, SD = 1.15), improved their attitudes toward diverse groups (M = 3.03, SD = 1.18), increased their awareness of personal values (M = 3.22, SD = 1.16), and heightened their awareness of the effects of their own prejudices (M = 3.21, SD = 1.21).

## Discussion

The present study investigated perceived diversity climate across three dimensions (age, gender, ethnicity), experiences and observations of discrimination, and various academic and psychosocial variables among students enrolled in health-related degree programs at German universities.

### Students’ perceived diversity climate in health-related degree programs

#### Perceived diversity climate

Overall, students in our sample perceived the diversity climate at their universities as moderately to highly positive across the examined diversity dimensions of age, ethnicity, and gender. Importantly, compared to students who did not personally experience discrimination, those who did reported significantly less favorable perceptions of the diversity climate across the assessed dimensions of gender, age, and ethnicity. The effect sizes ranged from medium (age) to large (gender, ethnicity), indicating substantial disparities in how students with and without experiences of discrimination perceive the diversity climate at their universities. This is consistent with previous findings showing that marginalized individuals often develop a heightened sensitivity to inequalities and injustices in academic settings due to their own repeated or cumulative experiences of discrimination. For instance, research from the United States indicates that students from minority groups perceive the diversity climate more negatively than students from majority groups. This includes, for example, perceived lower levels of inclusion, equal treatment, and institutional support for diversity [48, 64, 65]. Similarly, in a longitudinal survey of bachelor's students at a German university (*N* = 521), Hagelskamp et al. [4] found that students with a migration background experienced the diversity climate more negatively than students without a migration background. Their findings further suggest that students benefit when teachers explicitly value ethnic diversity and promote cooperation among ethnically diverse students, rather than ignoring differences in students' experiences and privileges, which has been described as using the construct of colorblindness. Moreover, it has been shown that perceptions of the diversity climate are associated with students’ stress, well-being, and academic outcomes [48, 66]. For instance, Denson and Chang [66] showed that the diversity climate correlates with students' academic self-efficacy and general academic skills in a large US student sample.

At an interpersonal level, i.e., the quality and frequency of interactions across diverse student populations, our findings suggest that participants generally perceived intergroup interactions among students as positive regardless of age, gender, or ethnicity. Notably, the consistently low ratings for support for positive interaction suggest that universities may fall short in actively fostering diversity and inclusion through institutional policies, teaching practices, and faculty engagement. Thus, positive intergroup relations at the interpersonal level are not necessarily accompanied by equally strong organizational efforts to promote diversity and inclusion. However, both levels are important in shaping students’ experiences of belonging, inclusion, and equity within educational settings. Byrd [48] demonstrated that when students perceive their university as fostering critical awareness of intergroup relations, privilege, and inequality and as actively valuing diversity and supporting intergroup contact, they report a stronger sense of belonging within the university. University lecturers play a key role in this process and can facilitate exchange and cooperation among diverse students through structured exercises and collaborative group work [3]. To effectively support positive interaction, students should be given adequate time to complete tasks, learning activities should be designed to explicitly benefit from diverse perspectives, and instructors should actively encourage group work, peer relationships, and student-led initiatives. Dogra et al. [23] emphasize the need to create learning environments in which students feel safe to articulate and critically examine differing viewpoints, supported by a shared language and structured forums that allow for the discussion of even sensitive or taboo topics.

In this study, perceptions of the gender-related diversity climate were rated more positively than those of the ethnicity- or age-related diversity climate. This may reflect the comparatively longer history of gender equality initiatives in Germany and correspondingly in the German school and higher education systems, including gender equality offices, formalized reporting structures, and increased public discourse on gender-inclusive language [67, 68). In contrast, ethnicity-related diversity climate perceptions were less favorable, which is consistent with prior findings indicating that progress in addressing different forms of inequality may be uneven and that racism and ethnic discrimination remain insufficiently addressed in German universities, particularly within predominantly *White* institutional contexts [24, 33]. Furthermore, these differences highlight the importance of examining diversity climate across multiple dimensions rather than treating diversity as a homogeneous construct. While this study examined the three dimensions of gender, age, and ethnicity, future studies should include additional intersecting dimensions of diversity, such as religion or sexual orientation.

#### Perceptions of diversity and equity-oriented beliefs

In this study, students who experienced personal discrimination reported significantly stronger diversity- and equity-oriented beliefs than those who did not. Although the effect size was small, this finding suggests that personal exposure to discrimination may increase awareness of structural inequalities and reinforce the perceived importance of diversity, equity and inclusion. Similar patterns have been observed in previous studies, in which members of marginalized groups expressed stronger support for diversity initiatives and anti-discrimination policies [69]. Although these findings are consistent with theoretical and empirical work, they should be interpreted in light of the study's cross-sectional design. The observed relationships reflect associations rather than causal effects, and the direction of these relationships cannot be determined.

#### Institutional infrastructure, diversity practices and perceived gaps

Beyond individual experiences, the study sheds light on students’ evaluations of institutional infrastructure and diversity practices. Across most diversity-related support services (e.g., for age, care work, ethnicity) and infrastructure (e.g., family and breastfeeding rooms, gender-neutral sanitary facilities, and rooms for religious practices), more than half of participants reported that these were not available or that they did not know whether they were. Moreover, we found limited use of available support services, which may be explained by the relatively homogeneous sample, as many participants may have less need for certain diversity-related support services. However, low utilization rates may also reflect a lack of awareness, concerns about stigma, or doubts regarding the effectiveness of such services [70–74]. The mere availability of diversity-related infrastructure may not be sufficient to ensure accessibility, equity, or trust. Instead, these services need to be actively communicated, visibly supported by institutional leadership, and embedded within a broader culture of inclusion. An additional particularly noteworthy finding is that nearly half of the respondents in this study indicated that lecturers were not sufficiently sensitive to discrimination or proactive in addressing it, and a majority reported inadequate representation of diverse groups in teaching materials.

### Discrimination experiences and their associations with academic and psychosocial outcomes

#### Discrimination

While the majority of participants (70%) did not report personal experiences of discrimination, nearly one-third (28%) indicated that they had experienced discrimination within the past two years in their university context, most commonly due to gender, physical appearance, mental health issues, or racial reasons. Moreover, almost half of all students had observed discrimination toward others, most commonly due to racial reasons, gender, physical appearance, or mental health issues. These findings are consistent with previous research showing a high prevalence of both witnessed and personally experienced discrimination in university and medical education settings. For instance, in a survey among students at German universities, 17.7% of whom were medical students, Führer et al. [38] found that 45% had witnessed discrimination in the university, while 28% reported first-hand experiences (68% repeatedly, 16% regularly) because of sex or gender (33%), non-German origin (13%) and a chronic illness (7%). They also reported that university lecturers were most often mentioned as perpetrators of discrimination. However, experiences of discrimination rarely occur along a single dimension of identity. Beyond overall prevalence, a growing body of literature emphasizes that discrimination increases when multiple diversity-related characteristics intersect. For example, Teshome et al. [75], who analyzed data from a cross sectional survey and retrospective cohort study more than 30,000 graduating medical students in the United States in 2016 and 2017, revealed that students with multiple marginalized identities, i.e., female, non-white, lesbian, gay, or bisexual, reported significantly more mistreatment and discrimination during medical school compared to male, white, and heterosexual students.

#### Associations with academic and psychosocial outcomes

The present findings demonstrate that experiences of discrimination are associated not only with perceptions of diversity climate but also with academic outcomes and student well-being. Although several group differences were statistically significant, the corresponding effect sizes varied considerably, suggesting their relevance and practical implications may differ across variables. Students who personally experienced discrimination reported significantly lower academic self-efficacy and study engagement, as well as a lower sense of belonging to their university. Crucially, among these outcomes, belonging to the university showed the largest effect size, highlighting its particular importance in relation to discriminatory experiences. Although general self-efficacy did not differ significantly between groups, domain-specific differences in academic self-efficacy suggest that discrimination is associated with confidence in academic contexts. Lower academic self-efficacy among students who experienced discrimination may reflect internalized doubt about one’s academic capabilities in environments perceived as unfair or exclusionary [76, 77]. This may be particularly relevant in health-related degree programs, which are often characterized by high performance pressure, competitive environments, and hierarchical structures [78–80].

Importantly, students who personally experienced discrimination also reported higher levels of general perceived stress in the present study, supporting previous findings that discrimination can be considered a chronic stressor with cumulative effects on well-being and functioning [48, 81, 82]. Surprisingly, differences in study-related stress did not reach statistical significance; however, the observed trend suggests an increased overall burden. These findings are consistent with previous research. For example, in a German study of 14,592 undergraduate and postgraduate students across a range of degree programs, Pilz González et al. [40] found that perceived discrimination, diverse or female gender, first-generation student status, and demanding family caregiving responsibilities were associated with poorer mental health outcomes.

## Limitations

Several limitations should be considered when interpreting the findings of this study.

First, several sampling-related limitations should be considered. This research used convenience and snowball sampling strategies, which may have introduced selection bias. Students with a particular interest in diversity-related topics, stronger opinions about diversity and inclusion, or personal experiences of discrimination may have been more motivated to participate than those who felt indifferent toward these topics. Likewise, students who had experienced particularly positive or negative diversity climates may have been more likely to participate. This could have affected the findings on the prevalence of experiences of discrimination, observations of discrimination, the perceived diversity climate or the perceived importance of diversity-related issues.

The sample cannot be considered representative of all students enrolled in health-related degree programs in Germany. Nevertheless, our findings are broadly consistent with those of previous nationwide studies. When interpreting the results, it is important to consider the specific characteristics of the German higher education system, especially the health-related education. As a result, the transferability of the findings to other national and educational contexts may be limited. International comparisons in the UNESCO Global Education Report [83] and the OECD Education Survey [84] highlighted the significant educational inequality in the high-income country Germany, often referred to as the “educational funnel”. In Germany, enrollment in higher education in general—and the choice of a health-related degree program in particular—depends on social determinants such as the parents’ ethnic or educational background. It has been repeatedly shown that, in Germany, students from non-academic backgrounds tend to prefer practical fields of study, while students from academic backgrounds are more likely to choose fields that are associated with high social status, such as medicine or law [85–87]. Compared to students in other fields of study, it is particularly common among students in medicine and dentistry for at least one parent to have completed a university degree [87]. Groene et al. [86] showed that students with migration backgrounds were underrepresented among medical applicants and students in Germany. With regard to social factors, the sample in this study can generally be considered relatively homogeneous and above average in terms of privilege. In contrast, the proportion of women in the study—which generally corresponds to the proportion of female students in health-related degree programs in Germany—is significantly higher than the 51% share of women in the total German student population [62]. Due to this distribution, results on gender-related (discrimination-related) experiences may not be representative of or applicable to other degree programs (e.g., those with significantly lower proportions of women, such as computer science or engineering).

The sample included students from a broad range of health-related degree programs, including medicine, psychology, public health, social work, nursing, and other health professions. While this broad recruitment strategy allowed us to collect comprehensive data, it may also have obscured important differences between disciplines. Educational contexts can vary with regard to curricula, professional cultures, student composition, exposure to diverse populations, and opportunities for intergroup contact, all of which may influence perceptions of the diversity climate, experiences and observations of discrimination, as well as psychosocial and academic variables.

Second, several measurement-related limitations should be acknowledged. All variables were assessed using self-report measures, which capture subjective perceptions rather than objective institutional indicators. Although anonymity likely reduced social desirability bias, individual differences in how individuals interpret discrimination, belonging, or institutional climate may have influenced responses. The measure of discrimination did not capture frequency or severity, which limits insight into cumulative exposure and its potential relationship to academic or psychosocial functioning. Moreover, while single-item measures help to reduce the survey length, they may capture complex constructs less comprehensively than multi-item scales (e.g., academic stress in the present study).

The adapted diversity climate scale focused only on three characteristics: age, gender, and ethnicity. While this approach ensured consistent measurement, it may underestimate the importance of other intersecting characteristics such as disability, socioeconomic background, caregiving responsibilities, religion, or sexual orientation. Future studies should adopt a broader, more intersectional operationalization of diversity. In addition, the stereotyping subscale demonstrated comparatively low internal consistency, particularly for age (α = 0.60) and gender (α = 0.66). The items may not have captured the construct of stereotyping as consistently as intended. Additionally, the validation of our adapted and extended version of the SCD-C was conducted in an unpublished study using the same dataset as the present study, as independent validation data were unavailable. Accordingly, findings from these measures should be interpreted with caution and, if possible, replicated using other measures.

Third, the cross-sectional design limits the ability to make causal claims. Although discriminatory experiences were associated with lower belonging, engagement, and academic self-efficacy, it remains unclear whether discrimination leads to poorer outcomes or whether students with higher stress or reduced well-being are more sensitive to discriminatory situations. Longitudinal or qualitative research would help clarify developmental processes and identify contextual factors that shape these experiences over time. Additionally, the study was not pre-registered, and no a priori sample size calculation or statistical power analysis was conducted.

Despite these limitations, the present study provides important national insights into students’ perceptions of the diversity climate, their experiences and observations of discrimination, and their psychosocial and academic well-being in health-related degree programs. The findings highlight the need for more representative, longitudinal, and intersectional research in this area, both in Germany and internationally.

## Implications for research, policy, and practice

The findings emphasize that promoting diversity in health-related higher education requires more than symbolic commitment or isolated initiatives. Universities in Germany and beyond should strengthen structural and curricular approaches to diversity, including mandatory diversity training for faculty and administrative staff, systematic integration of diversity-related content into curricula, and the active promotion of inclusive teaching practices. Given the strong association among personal experiences of discrimination, sense of belonging to the university, and academic outcomes, interventions aimed at fostering inclusion may also contribute to the well-being and academic success of diverse student populations.

The findings also suggest that diversity may be insufficiently embedded in curricula, accreditation processes, institutional monitoring and everyday teaching practices across German universities, despite increasing national and international calls for diversity-sensitive health professions education, such as published by the German Association for Medical Education (GMA) Committee on Cultural Competence and Global Health [24], by the Standing Committee of European Doctors [88], the International Association for Medical Education (AMEE; [22]), or by the International Association of Medical Science Educators (IAMSE; Powell & Linger [89]). To enhance diversity in medical education, lecturers are encouraged to critically review teaching materials and resources to ensure they reflect the full spectrum of diversity, thereby providing medical students with opportunities to engage with a genuinely diverse patient population [90]. According to Price et al. [91], ethnic minority faculty could serve as important role models and mentors for trainees, helping improve the diversity climate in academic medical settings. Research in other fields of higher education, such as teacher training, shows that stereotypical attitudes and discriminatory perceptions among students can be changed through targeted interventions that create awareness, build capacity and skills, and evoke emotional engagement [31]. In an umbrella review on cultural competency education for healthcare professionals including 12 systematic reviews published between 2005 and 2023, Lee et al. [92] found a wide range of delivery methods (e.g., lectures, interactive learning, immersive experiences, and digital tools such as virtual reality) resulting in predominantly positive learner outcomes (e.g., improved cultural knowledge and attitudes), whereas patient outcomes were evaluated less frequently and showed mixed results. In a longitudinal study spanning 10 years, Wingard et al. [93] demonstrated how regular institutional monitoring through faculty surveys, focused faculty development workshops based on issues identified in these surveys, and resulting institutional policy changes (e.g., Family Accommodations Policy) can interlink to systematically improve the diversity climate in academic medicine.

From a research perspective, the present analyses focused primarily on descriptive statistics and group comparisons between students with and without experiences of discrimination. While this approach aligned with the study's exploratory aims and predefined research questions, the dataset's richness would also enable more in-depth and complex analyses. These analyses will be explored in future publications and research. Moreover, future studies should adopt longitudinal designs to examine causal pathways among perceived diversity climate, experiences of discrimination, sense of belonging, academic outcomes, and students’ mental health and well-being. Although the study collected information on a broad range of sociodemographic and diversity-related characteristics, the analytical strategy did not explicitly examine intersectional patterns of discrimination. The present analyses may not fully capture the complexity of how overlapping identities influence experiences of discrimination, perceptions of diversity climate, and associated academic or psychosocial outcomes. Future research should adopt more intersectional approaches to investigate how combinations of identities shape students’ experiences within higher education institutions. Qualitative research could further deepen understanding of how students interpret and navigate diversity climates within specific institutional contexts [94, 95].

## Conclusion

In conclusion, this study shows that while the diversity climate across the characteristics of gender, age and ethnicity is perceived as moderately to highly positive, a substantial proportion of participants experience and observe discrimination in their institutional contexts. The findings also highlight that while diversity is broadly valued among the participating students in health-related degree programs in Germany, substantial challenges remain in translating these values into inclusive academic environments. Addressing discrimination and strengthening the diversity climate are not only matters of equity but also prerequisites for educating future healthcare professionals equipped to meet the needs of all patients in increasingly diverse societies.

## Supplementary Information

Supplementary Material 1

## Data Availability

The datasets generated and/or analyzed during the current study are not publicly available but are available from the corresponding author on reasonable request.
