# Supplementary material for: Diversity climate and discrimination at German universities: a cross-sectional study among students in health-related degree programs
Source: BMC Med Educ. 2026 Jul 7;26:1095. doi: 10.1186/s12909-026-09806-3 (PMC13344054; doi:10.1186/s12909-026-09806-3)
Supplement: Supplementary file 1 — Supplementary Material 1 [file 12909_2026_9806_MOESM1_ESM.docx]

**Appendix**

**Table A.** *Health impairments among the sample of students in health-related degree programs in Germany (N = 987)*

|  | *n* (%) |
| --- | --- |
| **Health impairment** |  |
| No | 497 (50.4) |
| Yes | 490 (49.6) |
| Physical impairments | 118 (12.0) |
| Mobility impairments (e.g., walking, standing, grasping, carrying) | 15 (1.5) |
| Hearing impairment / deafness | 9 (0.9) |
| Visual impairment / blindness | 94 (9.5) |
| Chronic impairments | 102 (10.3) |
| Specific learning disabilities | 51 (5.2) |
| Dyslexia | 33 (3.3) |
| Dyscalculia | 9 (0.9) |
| Speech impairment (e.g., stuttering) | 9 (0.9) |
| Psychological impairments | 292 (29.6) |
| Others | 144 (14.5) |

**Table B.** *Educational data of students in health-related degree programs in Germany (N = 987)*

|  | *n* (%) | *M (SD)* |
| --- | --- | --- |
| **Semester** |  | 4.95 (3.44) |
| **Highest professional qualification** |  |  |
| Doctorate/PhD/Habilitation | 8 (0.8) |  |
| Master’s degree | 36 (4.0) |  |
| Diplom degree | 6 (0.6) |  |
| State examination | 68 (7.6) |  |
| Bachelor’s degree | 229 (25.7) |  |
| Vocational college | 82 (8.3) |  |
| Apprenticeship | 48 (5.4) |  |
| Still in training | 359 (40.3) |  |
| Other | 44 (4.5) |  |
| **Academic performance (average grade)** |  | 1.9 (0.6) |
| Excellent | 208 (21.1) |  |
| Good | 355 (36.0) |  |
| Satisfactory | 88 (8.9) |  |
| Sufficient | 7 (0.7) |  |
| No mark received | 216 (21.9) |  |
| **Degree program**^1^ |  |  |
| Medical professions (e.g., human medicine, dental medicine) | 262 (26.5) |  |
| Health & rehabilitation sciences | 183 (18.5) |  |
| Psychology/psychotherapy | 153 (15.5) |  |
| Public Health | 73 (8.2) |  |
| Other therapeutic professions (e.g., occupational therapy, physiotherapy) | 66 (7.4) |  |
| Nursing and midwifery sciences | 63 (7.1) |  |
| Social professions (e.g., social work) | 53 (5.8) |  |
| Other degree programs | 73 (7.4) |  |
| **Federal state** |  |  |
| Baden-Württemberg | 116 (11.8) |  |
| Bavaria | 99 (10.0) |  |
| Berlin | 91 (9.2) |  |
| Brandenburg | 20 (2.0) |  |
| Bremen | 7 (0.7) |  |
| Hamburg | 44 (4.5) |  |
| Hesse | 90 (9.1) |  |
| Mecklenburg-Western Pomerania | 7 (0.7) |  |
| Lower Saxony | 89 (9.0) |  |
| North Rhine-Westphalia | 132 (13.4) |  |
| Rhineland-Palatinate | 23 (2.3) |  |
| Saarland | 1 (0.1) |  |
| Saxony | 41 (4.2) |  |
| Saxony-Anhalt | 55 (5.6) |  |
| Schleswig-Holstein | 58 (5.9) |  |
| Thuringia | 20 (2.0) |  |

**Note.** ^1^ = multiple answers possible

**Table C.** *Total and subscales of the SCD-C (Byrd, 2017, 2019)* *for assessing the diversity climate in health-related degree programs and higher-education institutions in Germany (N = 987)*

| (Sub-) Scale | Age *(M, SD)* | Ethnicity *(M, SD)* | Gender *(M, SD)* |
| --- | --- | --- | --- |
| Diversity climate | 3.91 (.59) | 3.63 (.65) | 3.83 (.59) |
| Quality of interaction | 3.80 (.69) | 3.78 (.74) | 4.05 (.68) |
| Frequency of interaction | 3.41 (.80) | 3.62 (.85) | 4.07 (.75) |
| Equal status | 4.17 (.77) | 4.01 (.90) | 4.13 (.82) |
| Support for positive interaction | 2.80 (1.17) | 3.04 (1.16) | 3.08 (1.14) |
| Stereotyping | 3.88 (.83) | 3.83 (.90) | 3.81 (.88) |
| **Note.** The scale ranges from 1 to 5, with higher values indicating better diversity climate. | | | |
